# Dexmedetomidine Reduced Cytokine Release during Postpartum Bleeding-Induced Multiple Organ Dysfunction Syndrome in Rats

**DOI:** 10.1155/2013/627831

**Published:** 2013-06-11

**Authors:** Liu Xianbao, Zhan Hong, Zeng Xu, Zhang Chunfang, Chen Dunjin

**Affiliations:** ^1^Department of Anesthesiology, Third Affiliated Hospital, Guangzhou Medical University, Guangzhou 510150, China; ^2^Department of Pathology, Detroit Medical Center, Harper University Hospital, School of Medicine, Wayne State University, 3990 John R, Detroit, MI 48201, USA; ^3^Department of Gynecology and Obstetrics, Third Affiliated Hospital, Guangzhou Medical University, Guangzhou 510150, China

## Abstract

Dexmedetomidine (DEX) is an **α**2-adrenergic agonist. It decreases the levels of norepinephrine release, resulting in a reduction of postsynaptic adrenergic activity. In the present study, the effects of DEX on postpartum bleeding-induced multiple organ dysfunction syndrome (BMODS) were studied in rats in which BMODS was induced by the combination of hypotension and clamping of the superior mesenteric artery. We evaluated the role of dexmedetomidine (DEX) in cytokine release during postpartum BMODS in rats. In summary, the present study demonstrated that DEX administration reduced IFN-r and IL-4 release and decreased lung injury during postpartum BMODS. It is possible that DEX administration decreased inflammatory cytokine production in BMODS by inhibiting inflammation and free radical release by leukocytes independent of the DEX dose.

## 1. Introduction

The most common cause of multiple organ dysfunction syndrome (MODS) in obstetric patients is postpartum bleeding followed by gestational hypertension syndrome [1, 2]. Bleeding-induced multiple organ dysfunction syndrome (BMODS) is a rapidly progressive disease that commonly occurs in critically ill obstetric patients with a high mortality rate. It is also one of the major causes of death in the maternity intensive care unit (ICU) [2]. BMODS induces diffuse ischemia and functional impairment in multiple organ systems and, in severe cases, can progress to diffuse intravascular coagulation (DIC) [3]. For this reason, patients with a history of heavy blood loss during delivery should be monitored in the ICU. The management of BMODS is challenging. Beyond prevention, BMODS treatment options are limited, and they are a popular topic for future clinical explorations. 

Dexmedetomidine (DEX) is an *α*2-adrenergic agonist. It selectively binds to presynaptic *α*2-adrenergic receptors (*α*2AR) on the locus coeruleus and decreases the levels of norepinephrine release, resulting in a reduction of postsynaptic adrenergic activity [4]. In the periphery, *α*2ARs are widely distributed in many organs, including the liver, kidney, pancreas, blood vessels, and platelets. The administration of DEX causes various effects in different organs [5, 6]. Currently, the primary clinical use of DEX is for its effects on the central nervous system, such as short-term sedation and antianxiety [7]. The pharmacological actions of DEX in other organs have not been fully evaluated in clinical patients. In animal studies, DEX has been shown to reduce mortality rates and inhibit inflammatory responses in endotoxemic rats without adverse effects on functions, such as respiration [8, 9]. However, the benefits of applying DEX to treat postpartum BMODS in maternity ICU patients have not been demonstrated due to the lack of a proper animal model [10]. In a pilot study, A. Sezer et al. found histopathological changes during sepsis-induced hypotension in rats [11]. In another study, liver and pancreatic dysfunctions were observed following the clamping of the superior mesenteric artery [12]. In the present study, the effects of DEX on postpartum BMODS were studied in rats in which BMODS was induced by the combination of hypotension and clamping of the superior mesenteric artery. We demonstrated that DEX administration reduced cytokine release and lung injury during postpartum BMODS in rats. 

## 2. Materials and Methods

### 2.1. Reagents

DEX was purchased from Hospira Inc. (Lake Forest, IL, USA). For reverse transcription polymerase chain reaction (RT-PCR) analysis, RNAiso Plus, and Taq DNA Polymerase were purchased from Takara Biotechnology Co., Ltd. (Dalian, China). The ReverTra Ace was purchased from Toyobo Biotech Co., Ltd. (Shanghai, China). Trizol reagent was purchased from Invitrogen (Carlsbad, CA, USA).

### 2.2. Animals

Sprague-Dawley (SD) rats (20 adult male and 40 adult female, 200–250 g) were purchased from the Animal Center of Guangdong Province. The rats were housed in the Guangzhou Animal Center in accordance with the specific pathogens animals standard with a 12 hr light/dark cycle. The room temperature was maintained at 22 ± 1°C. The rats had *ad libitum* access to food and drinking water. The animal studies in the present experiment were approved by the Animal Use and Care Committee of Guangzhou Medical University. The rats were handled following the National Institutes of Health guidelines. 

### 2.3. Experimental Groups

The male and female (1 : 2) rats were allowed to mate in the cage until the females were pregnant, as determined by detecting sperm in vaginal secretions using microscopy, at which point the females were separated from the males until delivery. Postpartum female rats were used within 24 hours after delivery. A total of 40 experimental female rats were randomly assigned into four groups, with 10 rats per group as follows. 

The Sham (S) group: catheters were placed in the right femoral artery and the left femoral vein;

 S+C group: bleeding-induced hypotension and clamping of the superior mesenteric artery were performed;
 S+C+D2.5 group: administration of DEX (2.5 *μ*g/kg/h) [13] to S+C rats;
 S+C+D5.0 group: administration of DEX (5.0 *μ*g/kg/h) [13] to S+C rats.

### 2.4. Experiment

For the S group, the rats were anesthetized by the intraperitoneal administration of 10% hydration chlorine aldehyde (0.4 mL/100 g). The rats were then placed supinely on an animal surgical table with a heating pad. The body temperature was maintained at 36–38°C throughout the experiment. To monitor blood pressure, a polyethylene catheter (PE-50) was inserted into the right femoral artery and connected to a blood pressure monitor. Another PE-50 was inserted into the left femoral vein for DEX administration. All of the catheters used in the present experiment were prepared with heparin to prevent blood clotting. For the S+C group, hypotension was induced by drawing blood from the right femoral artery into a glass syringe that was preloaded with heparin for temporary storage until the mean arterial pressure (MAP) reached the target level of 45–50 mmHg [14]. The MAP was constantly maintained at this low blood pressure level for 60 min by the withdrawal or reinfusion of storage blood via the right femoral artery. Thus, artificial bleeding and hypotension were established in the postpartum rats. Resuscitation was performed by re-infusing the full amount of blood that was previously taken from the rats to restore normal blood pressure. After a normal blood pressure was maintained for more than 30 min, the abdomen of the rat was opened under sterile conditions. The superior mesenteric artery was clamped for 60 min and then released. For the S+C+D2.5 and 5.0 groups, dexmedetomidine (2.5 *μ*g/kg/h and 5.0 *μ*g/kg/h, resp.) was administered via the left femoral vein for 4 hours.

After the previous experiment, the abdominal incision was sutured, and the catheters were removed. The rats were placed back in the cage and freely allowed to reach for food and drinking water. The rats were sacrificed 24 hours after the experiment. A 0.5-mL blood sample was taken from the abdominal aorta for immediate arterial blood gas level measurement. Another 0.7 mL of blood was taken for biochemical analysis. The left lung was harvested, immediately snap frozen in liquid nitrogen, and stored in the freezer for later histological and cytokine analyses. 

The changes in pulmonary, liver, and kidney functions were evaluated in BMODS-induced rats by measuring arterial blood gas, aminotransferase (AST), alanine aminotransferase (ALT), total bilirubin (TBIL), blood urea nitrogen (BUN), creatinine, and creatine phosphokinase (CPK). Morphological changes in the lung were evaluated using the index of quantitative assessment of histological lung injury (IQA) and alveolar interval thickness (AIT) [15, 16]. Histological sections from the lung were examined under the 200X power field. Lung injury was considered to be the presence of alveoli-containing erythrocytes or two or more neutrophils. The percentage of injured alveoli was counted from each field. The IQA was the mean percentage of injured alveoli from the 200X power field. The AIT from the lung was examined under the 200X power field using the IMS image analysis system (ShenTeng Information Technology Co., Ltd., Shanghai). Six fields were randomly selected from each power field and examined, making sure to avoid the bronchial and blood vessels. Six alveoli were randomly selected from each field for thickness measurements. The AIT was the mean thickness of the alveoli [15]. 

### 2.5. Reverse Transcription Polymerase Chain Reaction (RT-PCR)

RT-PCR was performed on tissue from the left lung. Total RNA was purified using the TRIzol kit (Takara Co., Ltd.). Residual genomic DNA was removed by incubation with RNase-free DNase. For the first strand cDNA synthesis, RNA (2 *μ*g) was converted to cDNA using superscript II reverse transcriptase (Invitrogen). The reaction mixture was inactivated by heating to 70°C for 15 min. One microliter of reaction mixture was amplified by Taq DNA polymerase (Takara Co., Ltd.) in a thermal cycler (GeneAmp, PCR system 2700, Applied Biosystems, Foster City, CA, USA). The first incubation was performed at 94°C for 3 min for initial denaturation, and the following steps were repeated 30 times: 30 s at 95°C (denaturation), 1 min (specific annealing temperature for each primer), and 1 min at 72°C (extension). The final incubation was at 72°C for 5 min (final extension). The sequences for the primers used in the present study are as follows: IL-4: sense 5′-TCCTTCACGGCAACAAGGAAC-3′ and antisense 5′-GTGAGTTCAGACCGCTGACA-3′ (predicted size: 168 bp, annealing temperature: 50°C); IFN-*γ*: sense 5′-GAACTGGCAAAAGGACGGTA-3′ and antisense 5′-GGATCTGTGGGTTGTT CACC-3′ (predicted size: 215 bp, annealing temperature: 49°C). The PCR products were size-fractioned by 1% agarose gel electrophoresis. After staining with ethidium bromide, the amplified DNA bands were analyzed with the image analysis software ScionImage (Scion Corp., Frederick, MD, USA).

### 2.6. Statistical Analysis

The statistical analysis was performed using Statistical Package from Social Sciences (SPSS) version 13.0 for Windows. All data were expressed as the mean ± SD. The Mann-Whitney *U* and *χ* ^2^ tests were used for the statistical analysis of the data among all the groups. *P* < 0.05 was considered to be statistically significant.

## 3. Results

### 3.1. Effects of DEX on Liver and Kidney Dysfunctions

Serum AST, ALT, BIL, BUN, Cr, and CPK were measured in the groups of 24 hours postpartum rats with BMODS (Table 1). There were significantly (*P* < 0.05) increased levels of AST, ALT, TBIL, BUN, Cr, and CPK in the S+C, S+C+D2.5, and S+C+D5.0 groups in comparison to the Sham group, indicating impaired liver and kidney functions in the postpartum rats with BMODS. There were no statistically significant differences (*P* > 0.05) between the S+C and S+C+D2.5 groups, the S+C and S+C+5.0 groups, and the S+C+D2.5 and S+C+5.0 groups, suggesting that DEX administration does not prevent liver and kidney damage in postpartum rats with BMODS.

### 3.2. Arterial Blood Gas (ABS) Analysis

ABS, including the potential of hydrogen (pH), partial pressure of carbon dioxide in arterial blood (PaCO2), partial pressure of oxygen in arterial blood (PaO2), and base excess (BE), was measured in postpartum rats with BMODS (Table 2). There were significant differences (*P* < 0.05) between the Sham, S+C, and S+C+D2.5/5.0 in the levels of PaCO2, PaO2, and BE, indicating impaired pulmonary function in postpartum rats with BMODS. There was no significant difference (*P* > 0.05) between S+C and S+C+D2.5 or between S+C and S+C+D5.0, suggesting that DEX administration does not improve ABS in postpartum rats with BMODS. 

### 3.3. Cytokine mRNA Expression

The mRNA expression of IFN-*γ* and IL-4 in the lung tissue of the S+C group was significantly (*P* < 0.05) higher than in that of the Sham group, indicating that an inflammatory response was elicited in postpartum rats with BMODS. The IFN-*γ*, IL-4, and IFN-*γ*/IL-4 mRNA ratios were significantly lower in S+C+D2.5 and S+C+D5.0 than in S+C, indicating reduced IFN-*γ* and IL-4 release upon DEX administration during postpartum BMODS. There were no statistically significant differences (*P* > 0.05) in IFN-*γ*, IL-4, and IFN-*γ*/IL-4 mRNA expressions between S+C+D2.5 and S+C+D5.0 (Table 3).

### 3.4. Morphological Evaluation of Lung Injury

**Figure 1:**
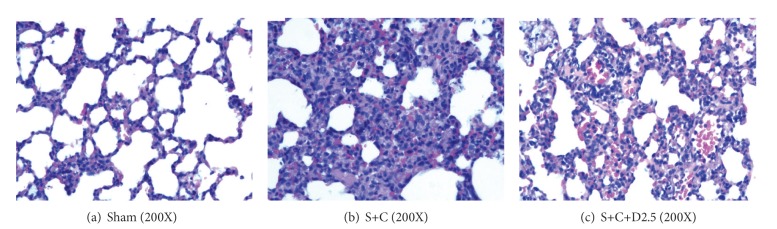
Histological sections from the lung were examined under the 200X power field. The IQA was the mean percentage of injured alveoli from the 200X power field. The AIT was examined under the 200X power field using the IMS image analysis system (ShenTeng Information Technology Co., Ltd., Shanghai). The AIT was the mean thickness of the alveoli.

In comparison to the Sham group (Figure 1(a)), lung sections from the S+C group (Figure 1(b)) showed acute injury; the interstitium was expanded by edema and inflammatory infiltrates that were composed of neutrophils, lymphocytes, and histiocytes. The alveoli were congestive and hemorrhagic. Some of the alveoli were collapsed. The epithelium was edematous and displayed a loss of cilia. The IQA and AIT were significantly higher in the S+C group compared to the Sham group (*P* < 0.05). The S+C+D2.5 and S+C+D5.0 groups showed less severe injury as compared to the S+C group, with significantly lower IQA and AIT values (*P* < 0.05) (Figure 1(c)). No significant differences were detected between the S+C+D2.5 and S+C+D5.0 groups (Table 4).

## 4. Discussion

In addition to treatment for sedation and antianxiety [17], DEX has been administered to hypertensive patients during surgery [18], suggesting a relaxing effect on peripheral vessels. DEX exerts its effects via the selective activation of *α*2AR. *α*2AR is distributed not only in the central and peripheral nervous system but also in multiple organ systems, where it exerts its effects on different physiological and pathological processes. For example, the clinical use of DEX has become popular in neurosurgery and heart surgery under extracorporeal circulation anesthesia [19, 20]. However, the effects of DEX on BMODS have not yet been evaluated. 

A small number of animal models are available for studying the pathogenesis of BMODS, but treatments have been largely unsuccessful, especially in patients with postpartum BMODS. It is difficult to study BMODS in human subjects because there are many factors involved in the pathogenesis and because there are a wide range of clinical presentations [21]. Therefore, the development of a successful animal model is a priority. In the present study, we established a BMODS model in postpartum rats. This animal model not only mimics postpartum bleeding-induced shock, but it also mimics reperfusion injury and endotoxicity. The majority of animal models for MODS are created by the external administration of endotoxin to induce hypotension [22]. Due to the wide range of individual sensitivities to endotoxin, many animals showed either a response too mild to induce hypotension or a response too strong, resulting in death of the animal. The level of hypotension and therefore the degree of injury are difficult to keep constant across groups. In the present study, hypotension was artificially controlled during the experiment. The time of clamping of the superior mesenteric artery was kept equal across the groups. Therefore, the level of damage was constant across the groups. The clamping of the superior mesenteric artery provided an ischemic and reperfusion process. This process induced endotoxin release rather than requiring external administration. Together with constant hypotension in rats, our animal model provides a reliable and constant level of damage across all groups for the study of BMODS. Our current BMODS model induced reperfusion injury. We observed inflammatory cytokine release in response to shock. Furthermore, clamping of the superior mesenteric artery induced multiple organ dysfunction syndrome, and it did not induce inflammatory cytokine release simply due to uncontrolled hemorrhagic shock; inflammatory cytokine release simply due to uncontrolled hemorrhagic shock differs from the response observed in patients undergoing postpartum hypotensive shock. Free radicals, calcium overload, and increased leukocytes play important roles in the process of ischemia-reperfusion injury. However, vasoactive peptides, regulatory peptides, inflammatory mediators, damaged cells, and metabolism also play important roles in the shock process. The results in present study showed that DEX administration decreased inflammatory cytokine release and damage in the lung in our current model of BMODS-induced reperfusion injury. The results showed that DEX may prevent organ damage through the inhibition of inflammation and free radical release from leukocytes. The specific mechanism of action of DEX in the BMODS model needs further research. According to the diagnosis criteria of MODS, damage of two or more organs must occur at the same time or sequentially within 24 hours of induction by pathogenic factors. Therefore, we sacrificed all of the rats 24 hours after the experiment. Liver, kidney, and lung dysfunctions were evident in the S+C group in the present study (Tables 1 and 2). However, there were no statistically significant differences between the S+C and S+C+D2.5 groups, S+C and S+C+5.0 groups, and S+C+D2.5 and S+C+5.0 groups, suggesting that DEX administration did not prevent liver and kidney damage in postpartum rats with BMODS. Several variables may explain these results, including that the time of DEX administration was too short, the specimen collection was too early (24 hours), or the dose of DEX administration was not suitable for use. At later time points of BMODS, biochemical markers may be more obviously altered, and increases in inflammatory cytokine release may be more apparent. Therefore, DEX administration may more clearly reduce biochemical markers and inflammatory cytokines at later time points. 

**Figure 2:**
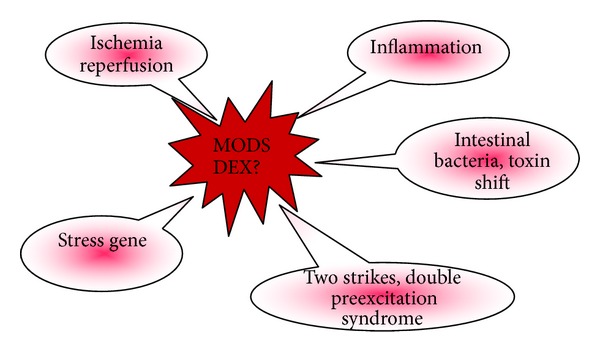
There have been several hypotheses regarding the mechanism of MODS (Figure 2), including ischemia reperfusion, inflammation, intestinal bacteria, toxin shifts, two strikes double preexcitation syndrome, and stress genes.

There have been several hypotheses regarding the mechanism of MODS (Figure 2), including ischemia reperfusion, inflammation, intestinal bacteria, toxin shifts, two strikes double preexcitation syndrome, and stress genes. However, none of these theories can fully explain the pathogenesis of MODS. The effects of DEX on the pathogenesis of MODS remain unclear. Regardless, the effect of DEX on inhibiting cytokine release was confirmed in this study. CD4+ T helper cells are divided into Th1 and Th2 subsets based on the cytokines they secrete [23]. Cytokines play an important role in lymphocyte development, maturation, differentiation, and activation. Th1 cells mainly secrete interleukin-2 (IL-2), IFN-*γ*, and tumor necrosis factor-*α* (TNF-*α*). They mediate cellular immunity and participate in late onset allergic reactions and inflammation. Th2 cells secrete IL-4, IL-5, IL-6, IL-9, IL-10, and IL-13 and are responsible for B lymphocyte proliferation, antibody production, immune tolerance, and humoral immunity [24, 25]. Therefore IFN-*γ* and IL-4 are the best respective markers for Th1 and Th2 cells, and they were chosen to evaluate the changes in immune function in postpartum rats with BMODS in the present study. DEX has been shown to inhibit cytokine secretion in many studies. Some reports have shown that DEX decreases cytokine secretion after endotoxin injection [26]. Other reports have demonstrated that DEX inhibits cytokine release in rats following endotoxin administration [22]. In mice, DEX administration led to a decrease in the total number of lung inflammatory cells, a reduction in the concentration of macrophage inflammatory protein-2 (MIP-2) and interleukin-β (IL-β), and a decrease in the ratio of dry/wet tissue in the lung [27]. To date, the mechanism underlying the role of DEX in the reduction of cytokine secretion is still a matter of debate. One group reported that DEX administration led to a reduced inflammatory response during the treatment of spinal cord injury, confirming the anti-inflammatory effects of DEX [28]. Another group reported that DEX protected nerve tissue from reperfusion-induced injury after -erm brain ischemia via reduced levels of TNF-*α* and decreased numbers of degenerative neurons in the hippocampus and dentate gyrus [29]. Our findings in the present study showed that DEX had strong anti-inflammatory effects on postpartum rats with BMODS. As shown in the S+C+D2.5 and S+C+D5.0 groups, DEX administration significantly reduced IFN-*γ* and IL-4. Because both IFN-*γ* and IL-4 represent humoral and cellular immunity, these findings suggested that both immune pathways were suppressed by DEX. Further, a decrease in the IFN-*γ*/IL-4 ratio suggested that DEX suppressed cellular immunity more than humoral immunity. There are few reports regarding a mechanism of DEX at the cellular level. Lower malondialdehyde (MDA) and nitric oxide (NO) levels and higher superoxide dismutase (SOD) and catalase (CAT) were observed in the hippocampus during reperfusion-induced injury in the ischemic rat brain after DEX administration [30]. DEX inhibited lipid peroxidation of the cell membrane in ischemia reperfusion models [31–33]. Further studies are required to address this issue in our model. Decreased immune function (both cellular and humoral) may induce septic shock. However, DEX administration did not lead to septic shock in our BMODS model, and this was confirmed by a report that DEX administration in early-stage sepsis patients decreased the mortality rate [27]. 

The decrease in cytokine release upon DEX treatment may protect organs from ischemic injuries. Clinically, DEX administration in combination with ketamine reduced mechanical ventilation-induced injury and inflammation in the lungs of endotoxemic rats [27]. In an animal model for spinal cord injury, the administration of DEX decreased edema and hemorrhage in gray matter without changing the number of neurons [34]. In the present study, DEX administration reduced inflammatory infiltration and edema in the lung, although no significant improvements were observed in ABS or liver and kidney function. This finding may provide a basis for additional management options for patients with BMODS. In current practice, BMODS patients are treated with mechanical approaches to prevent lung injury, including reducing the tidal volume of mechanical ventilation and positive pressure ventilation at the end of breath [35]. However, several studies have explored the combination of DEX and ketamine [36].

In summary, the present study demonstrated that DEX administration reduced IFN-*γ* and IL-4 release and decreased lung injury during postpartum BMODS. It is possible that DEX administration decreased inflammatory cytokine production in BMODS by inhibiting inflammation and free radical release by leukocytes independent of the DEX dose. 

## Figures and Tables

**Table 1:** Effects of DEX administration in liver, kidney functions during postpartum BMODS in rats.

Group	ALT (IU/L)	TBIL (umol/L)	BUN (mmol/L)	Cr (umol/L)	AST (IU/L)	CPK (IU/L)
Sham	46.1 ± 9.4	11.2 ± 1.9	2.6 ± 0.8	48.9 ± 4.7	168.3 ± 35.2	2103.4 ± 1045.6
S+C	383.7 ± 134.8^Δ^	56.6 ± 4.9^Δ^	5.5 ± 2.7^Δ^	196.6 ± 21.9^Δ^	611.3 ± 216.1^Δ^	9686.3 ± 1876.5^Δ^
S+C+D2.5	378.6 ± 139.3^Δ^	52.1 ± 6.9^Δ^	5.2 ± 2.5^Δ^	197.6 ± 23.6^Δ^	580.1 ± 230.0^Δ^	9597.0 ± 1879.4^Δ^
S+C+D5.0	376.1 ± 140.5^Δ^	53.5 ± 5.9^Δ^	5.5 ± 2.1^Δ^	195.9 ± 22.4^Δ^	601.4 ± 206.3^Δ^	9628.3 ± 1897.7^Δ^

^Δ^Compared to the Sham group *P* < 0.05; ^#^compared to the S+C group *P* < 0.05.

**Table 2:** Effects of DEX administration on arterial blood gas during postpartum BMODS in rats.

Group	PH	PaCO2 (mmHg)	PaO2 (mmHg)	BE (mmol/L)
Sham	7.40 ± 0.04	35.5 ± 4.2	92.2 ± 12.5	−1.0 ± 0.5
S+C	7.35 ± 0.07	43.7 ± 5.3^Δ^	65.6 ± 13.8^Δ^	−5.2 ± 0.4^Δ^
S+C+D2.5	7.32 ± 0.08	44.3 ± 4.5^Δ^	64.5 ± 14.5^Δ^	−4.8 ± 0.6^Δ^
S+C+D5.0	7.31 ± 0.07	43.5 ± 4.8^Δ^	65.8 ± 14.8^Δ^	−5.0 ± 0.7^Δ^

^Δ^Compared to the Sham group *P* < 0.05; ^#^compared to the S+C group *P* < 0.05.

**Table 3:** Effects of DEX administration on IFN-*γ*, IL-4, and IFN-*γ*/IL-4 mRNA expression during postpartum BMODS in rats.

Group	IFN-*γ*	IL-4	IFN-*γ*/IL-4
Sham	27.34 ± 1.56	23.38 ± 2.01	1.07 ± 0.04
S+C	34.51 ± 3.25^Δ^	32.35 ± 2.36^Δ^	1.06 ± 0.03^Δ^
S+C+2.5	20.27 ± 1.68^Δ#^	24.24 ± 2.02^#^	0.83 ± 0.02^Δ#^
S+C+5.0	19.06 ± 2.10^Δ#^	22.36 ± 1.31^#^	0.85 ± 0.03^Δ#^

^Δ^Compared to the Sham group *P* < 0.05; ^#^compared to the S+C group *P* < 0.05.

**Table 4:** The index of quantitative assessment of lung (IQA) and alveolar interval thickness (AIT).

Group	IQA (%)	AIT (*μ*m)
Sham	13.45 ± 3.84	6.9 ± 1.4
S+C	40.45 ± 4.24^Δ^	15.5 ±2.0^Δ#^
S+C+D2.5	30.67 ± 3.67^Δ#^	10.2 ± 2.3^Δ#^
S+C+D5.0	28.85 ± 3.45^Δ#^	9.8 ± 2.2^Δ#^

^Δ^Compared to the Sham group *P* < 0.05; ^#^compared to the S+C group *P* < 0.05.
